# The updated requirements and functional roles of dietary protein and amino acids in ducks: a comprehensive review

**DOI:** 10.1186/s40104-026-01366-2

**Published:** 2026-04-06

**Authors:** Yongbao Wu, Yong Jiang, Jing Tang, Shuisheng Hou, Zhiguo Wen

**Affiliations:** 1 Institute of Feed Research, Chinese Academy of Agricultural Sciences, Beijing, 100081 China; 2 Institute of Animal Sciences, Chinese Academy of Agricultural Sciences, Beijing, 100193 China; 3 College of Animal Science and Technology, Yangzhou University, Yangzhou, 225009 China

**Keywords:** Amino acid requirement, Duck nutrition, Functional role, Growth performance, Low-protein diet, Precision feeding

## Abstract

Waterfowl, primarily ducks, constitute a major commercial poultry species globally, their production efficiency is heavily influenced by dietary protein and amino acid intake. Although the National Research Council (NRC, Nutrient requirements of poultry, 1994) established fundamental recommendations for protein and amino acid requirements in ducks, tremendous improvements and changes (growth speed, rearing system, and dietary strategy) have been made in duck industry over the past decades. Consequently, these standards no longer adequately reflect the nutritional needs of modern duck production systems. This review presents updated estimates of dietary crude protein and key amino acid requirements, including methionine, lysine, threonine, tryptophan, and arginine, etc., which are essential for formulating precision diets to maximize growth performance, carcass yield in meat ducks, as well as reproductive performance in laying ducks and breeders. Furthermore, the functional roles of these amino acids as critical regulators of physiological processes are systematically examined, including lipid metabolism (methionine and threonine), intestinal barrier integrity (threonine), regulation of feed intake via nitric oxide (arginine), and the modulation of behavior and stress responses (tryptophan). The implementation of low-protein dietary strategies (crude protein levels as low as 15% for growing meat ducks, and 14.5% for laying ducks) is also discussed for maintaining performance while improving nitrogen utilization efficiency. The recent publication of updated Chinese national feeding standards for meat-type (GB/T 45103–2024) and laying ducks (GB/T 41189–2021) incorporates these modern research advances, representing a significant milestone and providing a modern framework to guide the global duck industries. In summary, this review highlights the urgent need to move beyond outdated nutritional standards and adopt modern, evidence-based amino acid requirements tailored to modern duck production. The integration of low-protein diet strategies along with a refined understanding of amino acid functions is crucial for optimizing duck production, enhancing nutrient efficiency, and reducing environmental impacts. Future research should focus on establishing the precise requirements for other essential amino acids and certain non-essential amino acids, and further elucidating how amino acids molecularly modulate gene expression, signaling pathways, and gut microbiota to develop innovative and sustainable nutritional strategies for ducks.

**Graphical Abstract:**

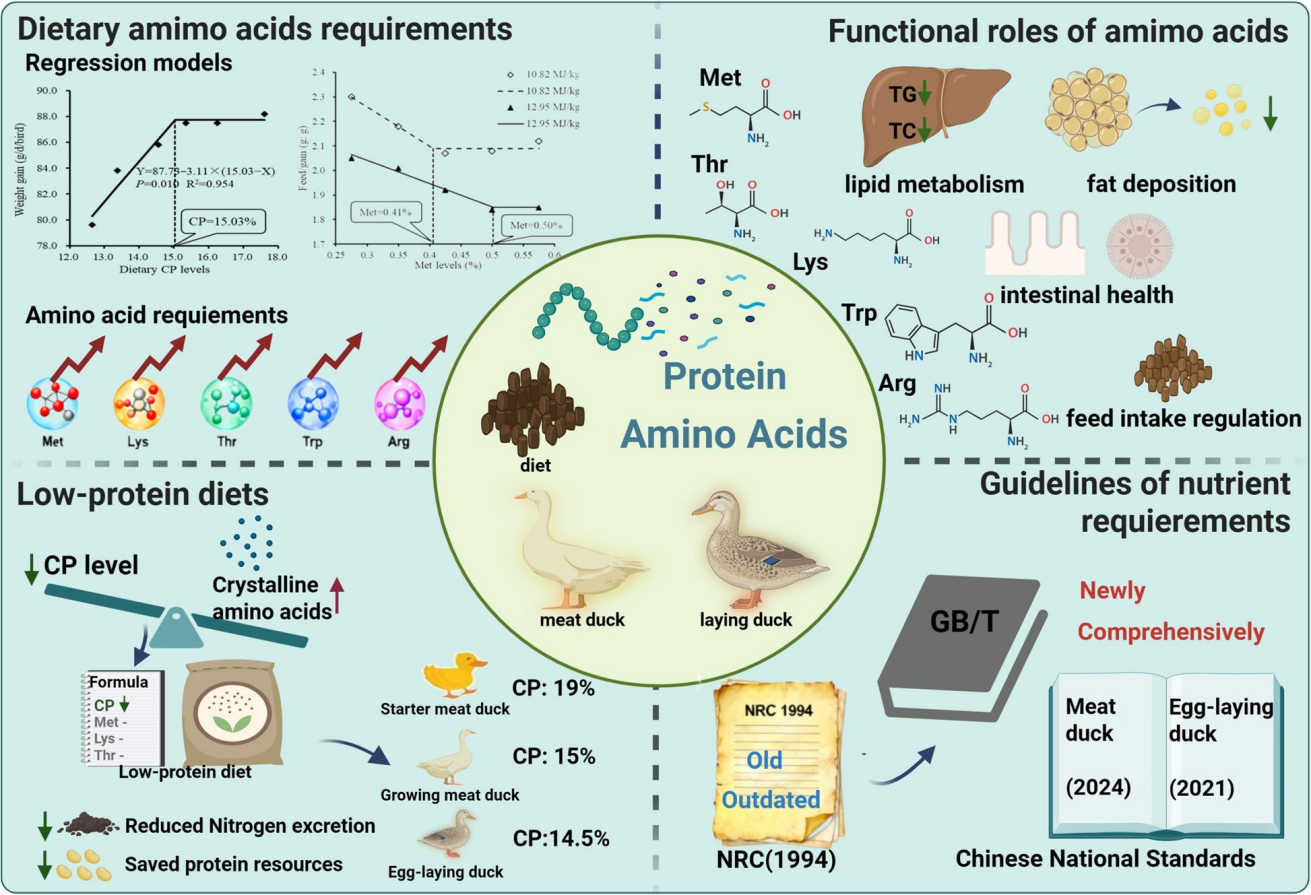

## Introduction

Waterfowl, primarily ducks, represent a major commercial poultry species globally. In recent decades, global consumption of duck meat has risen significantly, driven by its high nutritional value, which includes an optimal essential amino acid profile, a favorable polyunsaturated fatty acid composition, and a balanced ratio of omega-6 to omega-3 fatty acids [1]. According to the Food and Agriculture Organization of the United Nations, approximately 1.27 billion ducks were slaughtered in 2023, with Asia accounting for 90% of global production [2]. Although notable advancements have been made in feed nutrition and management practices, current dietary protein and amino acid requirement recommendations remain largely based on outdated standards, limiting their applicability to modern duck production systems [3].

Nutrient requirements define the minimum levels necessary to support normal growth and development during specific physiological stages. Diets formulated below these thresholds impair growth and developmental processes, whereas excessive nutrient supply results in inefficient resource utilization and increased production costs. Among these nutrients, essential amino acids are particularly critical because of their indispensable roles in protein synthesis and metabolic regulation in ducks. The nutrient requirement recommendations published by the National Research Council (NRC, 1994) [4] are largely outdated, as they were derived from a limited number of studies conducted several decades ago and therefore do not reflect the genetic advancements, intensified production systems, or modern management practices of contemporary duck breeds. Furthermore, although the NRC (1994) [4] provided requirement for most amino acids, several were either not explicitly defined (e.g., threonine (Thr)) or extrapolated from studies conducted in other poultry species like chickens (e.g., methionine (Met), lysine (Lys), etc.). Such extrapolations may be questionable given the well-documented physiological differences between ducks and chickens in digestive anatomy, amino acid digestibility, and nutrient retention efficiency [5]. Additionally, current amino acid recommendations fail to adequately consider key dietary factors such as energy and crude protein (CP) levels, as well as diet type. In duck diet formulation, for example, dietary energy should serve as the primary reference point, with other nutrients adjusted proportionally, because ducks regulate feed intake based on energy concentration [6, 7]. More importantly, tremendous genetic improvements have been achieved in duck production over the past decades, necessitating a reevaluation of amino acid requirements to align with the performance potential of modern strains. Compared with earlier lines, modern Pekin duck strains exhibit markedly greater feed intake, accelerated growth rate, and improved feed efficiency (e.g., live weight at 44 d, 2.95 vs. 3.50 kg; feed-to-gain ratio at 3.25 kg live weight, 2.65 vs. 1.85; market age, 49 d vs. 35 d) [8]. Therefore, it is imperative to update the established amino acid requirements and advance the understanding of their functional roles in ducks.

This review provides a comprehensive overview of research advances in dietary protein and amino acid nutrition for ducks over past decades, focusing on their requirements and physiological functions. By integrating the available evidence, this work aims to establish a scientific basis for refining amino acid recommendations and elucidating their critical roles in optimizing growth and productivity in ducks. The findings are expected to support the sustainable development of the duck industry by guiding future research and practical nutritional strategies.

## Dietary CP recommendation and low-protein diet in ducks

### Dietary CP requirement of ducks

Imbalanced dietary nutrition, resulting from either deficiency or excess, can adversely affect production performance in ducks. Among various nutritional factors, dietary CP level is a key determinant influencing duck growth and productivity. Protein requirements for ducks encompass needs for pre-maturity growth, egg production, as well as feather development and replacement. Inadequate CP intake or deficiencies in essential amino acids lead to reduced growth rate, decreased egg production, egg weight, and impaired hatchability. Recently, several studies have investigated dietary CP requirements in ducks (Table 1). For Pekin ducks aged 1 to 21 d, optimal dietary CP levels were estimated to be 20.63% and 23.25% based on daily gain and feed conversion efficiency, respectively [9]. Zeng et al. [11] examined the effects of dietary energy and protein levels on metabolizable energy and CP requirements in growing Pekin ducks, demonstrating that ducks fed a diet containing high metabolizable energy (ME, 13.75 MJ/kg) and high CP (19%, providing 1.21% standardized ileal digestible Lys) achieved the best growth performance in terms of daily weight gain and feed conversion efficiency. Using a 2 × 5 factorial design, Wang et al. [17] evaluated the effects of dietary CP levels and protease supplementation on growth performance, carcass traits, meat quality, and standardized ileal amino acid digestibility in Pekin ducks fed miscellaneous meal-based diets. Their results indicated that the optimal CP level was 17.02% without protease and could be reduced to 16.53% with protease supplementation, indicating that exogenous protease can partially reduce dietary CP requirements. Furthermore, Xia et al. [12] applied a 3 × 3 factorial design to evaluate three dietary energy levels (2,600, 2,500, and 2,400 kcal/kg) and three CP levels (19%, 18%, and 17%) in breeding laying ducks, concluding that a diet providing 2,451 kcal/kg of metabolizable energy and 19% CP, corresponding to a daily intake of 402 kcal of energy and 28.4 g of protein, optimized reproductive performance.

**Table 1:** Summary of the recommended CP requirement and low-protein diet in ducks during 2002–2025

Duck breeds	Age	Basal diet type	Model	Evaluation indicator	ME level, MJ/kg	CP level	Reference
Studies on crude protein requirement							
Pekin duck	1–21 d	Corn, wheat, soybean meal, fish meal	Linear or quadratic broken line	ADG, F/G	13.40	20.63%–23.25%	[9]
	15–40 d	Corn, soybean meal, wheat flour, rice bran	-	F/G	12.35	16.5%	[10]
	15–35 d	Corn, soybean meal, wheat bakery meal	-	ADG, F/G	13.75	19.0%	[11]
Longyan laying breeder	29–45 weeks	Corn, soybean meal, wheat middling, corn gluten meal	Quadratic regression	FCR, reproductive performance	10.26	19.0%	[12]
Studies on low-protein diet							
Pekin duck	1–19 d	Corn, soybean meal	Linear broken line	F/G	12.05	19.68%	[13]
	15–35 d	Corn, soybean meal	Linear broken line	F/G, abdominal fat yield	12.31	14.81% –14.94%	[14]
	21–42 d	Corn, soybean meal, rapeseed meal	Linear broken line	ADG, F/G	12.16	15.03% –15.24%	[15]
Jingjiang laying ducks	50–61 weeks	Corn, soybean meal, wheat flour, rice bran	-	Laying performance, egg quality	11.1	14.5%	[16]

"-" means no model used

*ADG* Average daily gain, *F/G* Feed to gain ratio, *FCR* Feed conversion rate

### Low-protein diet in ducks

The adoption of low-protein diets has emerged as a promising strategy in intensive livestock and poultry production to conserve feed resources and mitigate environmental impacts. Appropriately reducing dietary protein levels can improve growth performance in meat ducks by enhancing the protein utilization and deposition efficiency, thereby increasing feed conversion efficiency, provided that diets contain adequate and balanced amino acid profiles. Supplementation of low-protein diets with synthetic amino acids, while maintaining sufficient total nitrogen and energy supply, has been shown to exert no adverse effects on growth performance or carcass traits in meat ducks (Table 1). Previous studies have demonstrated that a moderate reduction in dietary CP levels does not significantly impair growth or slaughter performance, and that dietary CP levels can be reduced to 19.68% for starter Pekin ducks [13] and 14.94% for growing Pekin ducks [14]. Similarly, Wu et al. [15] reported that reducing dietary CP levels did not significantly affect growth performance or carcass characteristics of Pekin ducks reared in multi-tier cage systems. Notably, lower CP diets markedly reduced protein intake, nitrogen intake, and nitrogen excretion, while simultaneously increasing the apparent digestibility of energy, protein, and dry matter. These findings indicate that the CP level can be lowered to approximately 15% when essential amino acid requirements are adequately met through synthetic amino acid supplementation [15]. In laying ducks, Zhang et al. [16] indicated that dietary CP could be reduced to 14.5% for up to 12 weeks without compromising egg production or egg quality when supplemented with amino acids, based on comprehensive evaluations of production performance, egg quality, serum biochemical indices, and follicular development in Jingjiang ducks. Since protein nutrition fundamentally depends on amino acids or peptides, supplementation of low-protein diets with crystalline amino acids (e.g., DL-Met [18]) or exogenous protease [19, 20] can significantly enhance growth performance, and improve intestinal health. Although such low-protein diets may not support maximal growth under all conditions, they effectively reduce nitrogen excretion and contribute to mitigating environmental pollution [15].

It has been estimated that reducing dietary CP level by 1% could decrease soybean meal inclusion by approximately 3% to 4%. The first national standard in this field, “*Specification of practice for the diversified diets with low protein and low soybean meal for meat ducks ”* [21], has been officially established. This guideline plays a crucial role in conserving protein feed resources, promoting the efficient utilization of unconventional feed ingredients, reducing nitrogen emissions from duck production systems, and ensuring the quality, safety, and steady stable of duck products. Although low-protein diets balanced with crystalline amino acids offer clear benefits in improving nitrogen utilization efficiency and reducing environmental emissions, their successful application requires a high degree of formulation precision [22]. Accurate digestible amino acid values for feed ingredients are essential, as small deviations may adversely affect performance under reduced crude protein levels. In addition, the economic feasibility of extensive crystalline amino acid supplementation and its cost sensitivity under commercial conditions remain important considerations.

## Dietary amino acid recommendation and importance for ducks

### Methionine

Met is the first limiting-amino acid in conventional corn-soybean meal-based diets for ducks [23], and plays a vital role in protein synthesis and metabolic regulation. Optimal dietary Met supplementation has been shown to increase the growth performance, carcass yield and egg production in ducks [24, 25]. However, the Met requirement of NRC (1994) [4] recommendation is outdated, and the data was derived from a single study conducted in Muscovy ducks. Hence, dietary Met requirements should be re-evaluated to better reflect the needs of different duck types, particularly modern meat-type and egg-type ducks (Table 2). In our previous study, the dietary Met requirement of male Pekin ducks from 21 to 49 d of age (growing meat-type duck) were estimated to be 0.377% for maximizing weight gain and 0.379% for optimizing breast meat yield, respectively [24]. In contrast, the greater Met requirements for growing Pekin ducks were reported by Zeng et al. [28], who found that the requirement for optimal feather coverage exceeded those for body weight, feed/gain, and breast meat yield. These discrepancies are likely attributable to differences in experimental conditions, including basal diet composition and duck strain. Among these factors, dietary ME level is considered particularly important because of its influence on feed intake. The higher dietary energy levels typically necessitate greater amino acid concentrations to compensate for reduced feed intake for ducks [7]. Hence, a subsequent study was conducted to investigate the impact of dietary ME level on Met requirement of growing Pekin ducks fed a conventional corn-soymeal diet from 15 to 42 d of age [6], which observed an interaction effect on feed-to-gain ratio between dietary ME and supplemental Met level. Based on broken-line regression models, the estimated dietary Met requirements of growing Pekin ducks for optimal feed-to-gain ratio differed when expressed as a percentage of the diet at 10.82 and 12.95 MJ/kg of ME (0.406% vs. 0.502%), whereas no difference (0.376 vs. 0.388 g/MJ) was observed when requirements were expressed as g/MJ ME of diet in both dietary ME levels. It indicated that although absolute dietary Met levels are influenced by dietary ME, the Met/ME ratio remains relatively constant in corn-soymeal diet of growing Pekin ducks [6]. In breeding ducks, Ruan et al. [25] investigated the effects of dietary Met levels on reproductive performance, antioxidant capacity, and the expression of genes associated to ovalbumin and antioxidant enzymes. Their results demonstrated that a diet containing 0.40% Met optimized hepatic glutathione peroxidase 1 (GPX1) and nuclear factor erythroid 2-related factor 2 (Nrf2) expression, maximized glutathione peroxidase activity, and minimized lipid peroxidation [25].

**Table 2:** Summary of the recommended requirement of amino acids in ducks during 2002–2025

Amino acids	Duck breed	Age	Basal diet type	Energy level, MJ/kg	CP level	Dietary amino acid levels	Model	Evaluation indicator	Requirement	Reference
Met	Pekin duck	1–21 d	Corn, peanut meal	12.32	20.1%	Met: 0.285%, 0.385%, 0.485%, 0.585%, 0.685%,	Quadratic regression	ADG	0.481%	[26]
		1–14 d	Corn, peanut meal, soybean meal	12.45	20.26%	Met: 0.26%, 0.46%, 0.66%	-	BW	0.46%	[27]
		21–49 d	Corn, peanut meal, soybean meal	12.18	17%	Met: 0.20%, 0.275%, 0.35%, 0.425%, 0.50%, 0.575%	Quadratic regression	ADG	0.377%	[24]
								BMY	0.379%	
		15–42 d	Corn, soybean meal	10.82	16.86%	Met: 0.275%, 0.35%, 0.425%, 0.50%,0.575%	Linear broken line	F/G	0.406%	[6]
				12.95	16.96%	Met: 0.277%, 0.352%, 0.427%, 0.502%,0.577%			0.502%	
		15–28 d	Corn, soybean meal, mixed meal	13.37	18.08%−18.42%	Met: 0.30%, 0.39%, 0.45%, 0.56%, 0.68%	Quadratic broken line	BW	0.510%	[28]
								BMY	0.445%	
								FPWFL	0.404%	
							Quadratic regression	BW	0.606%	
								BMY	0.576%	
								FPWFL	0.559%	
		15–35 d	Corn, soybean meal, mixed meal	13.37	18.08%−18.42%	Met: 0.30%, 0.39%, 0.45%, 0.56%, 0.68%	Quadratic broken line	BW	0.468%,	
								BMY	0.408%	
								FC	0.484%	
							Quadratic regression	BW	0.605%,	
								BMY	0.564%,	
								FC	0.612%	
	Longyan duck breeder	19–43 weeks	Maize, mazie starch, wheat bran, soyabean meal, peanut meal	10.46	17.17%	Met: 2.00, 2.75, 3.50, 4.25, 5.00, 5.75 g/kg	Quadratic regression	Egg weight	0.46%	[25]
								Albumen weight	0.49%	
								Egg mass	0.44%	
								FCR	0.48%	
								Hatchability	0.40%	
								Hepatic GPX activity	0.40%	
								Hepatic *GPX1* expression	0.40%	
								Hepatic *Nrf2* expression	0.40%	
	Longyan laying duck	19–47 weeks	Maize, soybean meal	10.46	17.0%	Met: 2.5, 3.0, 3.5, 4.0, 4.5, 5.0 g/kg	Quadratic broken line	Egg weight,	686 mg/bird/d	[29]
								Egg mass,	661 mg/bird/d	
								FCR	658 mg/bird/d	
								Albumen weight	731 mg/bird/d	
Met + Cys	Korean native duck	1–21 d	Wheat, maize, soybean	13.5–13.6	17.9%−18.2%	TSAA: 0.62%, 0.65%, 0.68%, 0.71%, 0.74%, 0.77%,0.80%	Linear or quadratic broken line	ADG	0.70%	[30]
								ADFI	0.66%	
								F/G	0.70%	
Lys	Pekin duck	1–14 d	Corn, peanut meal, soybean meal	12.45	20.26%	Lys: 0.95%, 1.10%, 1.25%	-	BW	1.10%	[27]
		1–21 d	Maize, wheat, wheat gluten, wheat bran, soybean meal	12.67	22.4%	Lys: 0.76%, 0.86%, 0.96%, 1.06%, 1.16%, 1.26%	Modified Gauss–Newton iterative algorithm	ADG	1.17%	[31]
								F/G	1.06%	
		22–49 d		13.30	21.5%	Lys: 0.62%, 0.72%, 0.82%, 0.92%, 1.02%, 1.12%		ADG	0.87%	
								F/G	0.82%	
								BMY	1.00%	
		1–21 d	Corn, DDGS, soybean meal, peanut meal	11.51	19.65%	Lys: 0.70%, 0.79%, 0.90%, 1.01%, 1.09%, 1.20%	Linear broken line	ADG	0.94%	[7]
				12.76	19.58%	Lys: 0.70%, 0.80%, 0.89%, 0.98%, 1.10%, 1.19%			0.98%	
		7–21 d	Corn, soybean meal, peanut meal	12.13	20.30%	Lys: 0.65%, 0.80%, 0.95%, 1.10%, 1.25%	Linear broken-line model	ADG	0.84%	[32]
								F/G	0.90%	
								BMY	0.98%	
	Korean native duck	1–21 d	Wheat, corn, soybean meal	13.40	12.2%−18.2%	DLys: 0.44%, 0.53%, 0.62%, 0.71%, 0.80%, 0.89%, 0.98%, 1.07%	Linear or quadratic broken line	BW	0.71%	[33]
								ADG	0.74%	
								ADFI	0.65%	
								FCR	1.01%	
	Cherry Valley ducks	1–14 d	Corn, wheat bran, soybean meal, rapeseed meal	12.18	20.10%	DLys: 0.80%, 0.88%, 0.96%, 1.04%, 1.12%	Quadratic regression	ADG	0.948%	[34]
								F/G	0.986%	
								BMY	0.961%	
		15–35 d		12.30	16.50%	DLys: 0.60%, 0.68%, 0.76%, 0.84%, 0.92%		ADG	0.758%	
								F/G	0.792%	
								BMY	0.761%	
	Longyan laying duck	22–38 weeks	Soybean meal, wheat bran, corn gluten	10.47	17.0%	Lys: 0.75%, 0.80%, 0.85%, 0.90%, 0.95%	-	Egg weight	0.80%	[35]
	Longyan duck breeder	19–45 weeks	Corn, wheat bran, soybean meal, peanut meal	10.46	17.25%	Lys: 0.64%, 0.72%, 0.80%, 0.88%, 0.96%, 1.04%	Quadratic regression	Egg production	0.88%	[36]
								Egg weight	0.86%	
								Egg mass	0.90%	
								FCR	0.91%	
								Hatchability	0.91%	
								Nitrogen deposition	0.95%	
Thr	Pekin duck	1–14 d	Corn, soybean meal, corn starch	12.78	20.65%−20.98%	Thr: 0.60%, 0.75%, 0.89%, 0.95%, 1.01%, 1.09%	Quadratic broken-line	ADG	0.86%	[37]
								F/G	0.92%	
								BMY	0.95%	
							Quadratic regression	ADG	0.94%	
								F/G	0.98%	
								BMY	0.96%	
		1–14 d	Corn, corn gluten meal, wheat, peanut meal	12.14	16.0%	Added Thr: 0, 0.07%, 0.14%, 0.21%, 0.28%	Linear broken line	ADG	0.56%	[38]
					17.5%				0.61%	
					19.0%				0.60%	
					20.5%				0.63%	
					22.0%				0.67%	
		1–21 d	Corn, corn starch,soybean meal, peanut meal	12.64	18.98%	Thr: 0.50%, 0.58%, 0.66%, 0.74%, 0.82%	Quadratic broken-line	ADG	0.67%	[39]
		1–21 d	Corn, wheat, peanut meal	12.10	20.89%	Added Thr: 0, 0.06%, 0.12%, 0.18%, 0.24%, 0.30%	Quadratic broken-line	ADG	0.61%	[40]
				12.20	17.78%				0.56%	
		1–21 d	Corn, corn gluten meal, wheat, peanut meal	12.14	19.0%	Added Thr: 0, 0.07%, 0.14%, 0.21%, 0.28%	Quadratic broken-line	ADG	0.66%	[41]
					22.0%				0.70%	
					19.0%			BMY	0.67%	
					22.0%				0.72%	
		15–35 d	Corn, soybean meal, bakery meal, wheat middling, animal-vegetable fat blend	-	16.39%−18.46%	Thr: 0.57%, 0.60%, 0.64%, 0.72%, 0.80%	Quadratic broken-line	ADG	0.71%	[42]
								F/G	0.69%	
								BMY	0.69%	
							Quadratic regression	ADG	0.76%	
								F/G	0.74%	
								BMY	0.73%	
	Longyan laying duck	17–45 weeks	Corn, wheat, wheat bran, peanut meal	10.46	16.0%	Thr: 0.39%, 0.44%, 0.49%, 0.54%, 0.59%, 0.64%	Quadratic broken-line regression	Egg production	0.57%	[43]
								Egg mass	0.57%	
								FCR	0.57%	
								Egg weight	0.58%	
Trp	Pekin duck	1–21 d	Corn, corn gluten meal, peanut meal	12.75	18.1%	Trp: 0.132%, 0.167%, 0.202%, 0.237%, 0.272%	Linear broken-line regression	ADG	0.168%−0.242%	[44]
	Longyan laying duck	22–37 weeks	Corn, soybean meal, wheat bran, corn gluten meal	10.70–10.81	16.9%−17.2%	Trp: 0.09%, 0.192%, 0.310%, 0.392%, 0.489%, 0.579%	Quadratic regression	Egg production	0.314%	[45]
								Egg mass	0.293%	
								FCR	0.292%	
Arg	Pekin duck	1–21d	Corn grain, corn gluten meal	12.89	22.02%	Arg: 0.82%, 0.93%, 1.04%, 1.15%, 1.26%, 1.37%, 1.48%, 1.59%, 1.70%	Quadratic broken-line regression	ADG	0.95%	[46]
								F/G	1.16%	
								BMY	0.99%	
	Longyan laying duck	17–31 weeks	Corn, wheat middling, DDGS, corn gluten meal	10.4	17.0%	Arg: 0.66%, 0.86%, 1.06%, 1.26%, 1.46%	Quadratic regression	Yolk percentage	1.06%	[47]
								SYF number	1.13%	
								SYF weight	1.22%	
								SYF weight/ovarian weight	1.11%	

*Met* Methionine, *Cys* Cysteine, *Lys* Lysine, *Thr* Threonine, *Trp* Tryptophan,* Arg* Arginine, *TSAA* Total sulfur-containing amino acid, *ADG* Average daily gain, *BMY* Breast meat yield, *BMW* Breast meat yield, *BW* Body weight, *F/G* Feed to gain ratio; *FPWFL* Fourth primary feather length, *FC* Feather coverage, *FCR* Feed conversion ratio, *GPX* Glutathione peroxidase, *SYF* Secondary yolk follicle

“-” means no model used

Over recent decades, numerous studies have investigated the functional roles of Met in the growth and productivity of meat and laying ducks. Dietary Met deficiency has consistently been shown to impair growth performance and carcass yield in meat ducks [24, 48]. Conversely, previous studies have reported a quadratic response of weight gain to increasing dietary Met levels in corn-peanut meal-based diets fed to ducks from hatch to 21 days or from 21 to 49 days of age, with performance initially improving and subsequently declining at excessive Met levels [6, 24]. Moreover, Met deficiency in diets could decrease breast and leg meat yield, and resulted in excess abdominal fat deposition in Pekin ducks [6, 24, 48, 49]. Mechanistically, dietary Met deficiency affected expression of genes and proteins associated with lipid metabolism and stunted growth of Pekin ducks. Additionally, decreased hepatic albumin expressions and downregulation of lipolytic genes and proteins in abdominal fat may compromise fatty acid transport, contributing to excessive fat accumulation [48]. In laying breeder ducks, dietary Met deficiency induces a reduction in duckling hatching weight, alterations lipid and liver metabolism in offspring [50], and these deficiency symptoms could be counteracted via dietary Met supplementation in laying duck breeder [25]. Such benefits are partly attributed to enhanced intestinal development, including increased ileal villus height and improved nutrient absorption [51, 52]. Furthermore, dietary Met can enhance antioxidant capacity by increasing glutathione (GSH) content and glutathione peroxidase (GPx) activity, while reducing malondialdehyde (MDA) levels and protein carbonylation. Consistently, dietary Met supplementation enhanced the antioxidant properties of the pectoralis major muscle in meat ducks [53]. At the molecular level, the expression of genes related to antioxidant defense in the liver of laying breeder ducks and in the brain of their offspring increased quadratically with increasing dietary Met supplementation [25]. In addition, dietary Met deficiency, given its role as a primary methyl donor, upregulates the expression of methionine synthase (MS) and betaine-homocysteine methyltransferase (BHMT), enhancing the transcription of enzymes involved in homocysteine remethylation as a compensatory mechanism to promote endogenous Met synthesis [54].

The stunted growth and decreased carcass yield by dietary Met deficiency of ducks could be counteracted via addition of synthesized DL-Met or its hydroxy analogue (2-hydroxy-4-methylthiobutanoic acid; HMTBA) in diets [55]. Recent studies have demonstrated comparable bio-efficacy between DL-Met and HMTBA in Pekin ducks, with relative efficacy values of 101% during the 1 to 21 days of age [56] and 99% during the 1 to 42 days of age [53], respectively. Notably, HMTBA has been shown to confer greater antioxidant capacity in the pectoralis major muscle compared with DL-Met [53]. These findings suggest that although DL-Met and HMTBA are nutritionally equivalent in supporting growth performance and carcass traits in ducks, HMTBA may provide additional benefits by enhancing oxidative stress resistance and overall health status in ducks. Chemically synthesized DL-Met, which is commonly supplemented in feed formulation, is a racemic mixture containing equal proportions of D- and L-Met. While L-Met can be directly incorporated into body protein with full efficiency, D-Met must first be converted to the L-Met prior to utilization, a process that may reduce its overall absorption and metabolic efficiency. Recently, crystalline L-Met has become commercially available as a novel Met source for animal diets. Studies have shown that the bio-efficacy of L-Met relative to DL-Met ranges from 120% to 140% for growth performance in Pekin ducks [52], indicating that L-Met is utilized approximately 1.2- to 1.4-fold more efficiently than DL-Met. Given its high utilization efficiency and increasingly competitive production costs, the inclusion of crystalline L-Met in duck diets may represent a rational and cost-effective nutritional strategy. Nevertheless, regardless of the Met source used, appropriate supplementation is critical, as excessive Met intake has been shown to be toxic and to depress growth performance in ducks [55, 57]. Excessive supplementation with DL-Met, HMTBA [53] or L-Met [57] adversely affected growth performance in Pekin ducks, with the total tolerable upper limit of dietary Met estimated to be approximately 0.90% for starter Pekin ducks [57]. The growth depression associated with excessive Met intake may be related to elevated plasma homocysteine levels, although the underlying mechanisms remain to be fully elucidated [55].

### Lysine

As the second-limiting amino acid in ducks, Lys requirements have been extensively evaluated. The NRC (1994) [4] recommendation provides Lys levels of 0.90% (hatch to 14 days of age) and 0.65% (15 to 49 days of age) for Pekin ducks. However, these values were derived from a limited number of early studies (Table 3). Subsequent studies consistently suggested higher Lys requirements for ducks when estimated using various response models (Table 2). For example, Bons et al. estimated Lys requirements for average daily gain and feed-to-gain ratio of 1.17% and 1.06% for Pekin ducks from hatch to 21 days of age, and 0.87% and 0.82% for ducks from 22 to 49 days of age, respectively [31]. Similarly, Xie et al. [32] estimated Lys requirements to be 0.84% for weight gain, 0.90% for feed-to-gain ratio, and 0.98% for breast meat yield in Pekin ducks from 7 to 21 days of age. Zhou et al. [34] further demonstrated that Lys requirements for weight gain, feed-to-gain ratio, and breast meat yield were 0.948%, 0.986%, and 0.961% for ducks from 1 to 14 days of age, and 0.758%, 0.792%, and 0.761% for ducks from 15 to 35 days of age, respectively. These estimates generally exceed the NRC (1994) [4] recommendations. The variability in reported Lys requirements among studies can likely be explained by differences in response criteria, which are influenced by environmental conditions, interactions with other limiting nutrients, and genetic potential [58, 59]. For instance, Lys requirement for weight gain was higher at a dietary ME level of 12.76 MJ/kg (0.98%) than at 11.51 MJ/kg (0.94%), reflecting compensation for reduced feed intake associated with higher-energy diets [7]. In addition to growing ducks, Lys requirements have also been reported to be approximately 0.80% for laying ducks aged 22 to 38 weeks [35], and 0.86% to 0.91% for breeding ducks [36].

**Table 3:** Dietary energy, crude protein and amino acid requirements for ducks as recommended by NRC (1994) [4]

Nutrients	Starter (0–2 weeks)	Grower (2–7 weeks)	Breeder
ME, kcal/kg	2,900	3,000	3,000
CP, %	22	16	15
Lys, %	0.90	0.65	0.60
Met, %	0.40	0.30	0.27
Met + Cys, %	0.70	0.55	0.50
Trp, %	0.23	0.17	0.14
Arg, %	1.1	1.0	-
Val, %	0.78	0.56	0.47
Ile, %	0.63	0.46	0.38
Leu, %	1.26	0.91	0.76

*ME* Metabolizable energy, *CP* Crude protein, *Met* Methionine, *Cys* Cysteine, *Lys* Lysine, *Thr* Threonine, *Trp* Tryptophan, *Arg* Arginine, *Val V*aline, *Ile* Isoleucine, *Leu* Leucine

Lys is a fundamental structural component of proteins and plays essential roles in the synthesis of skeletal muscle, enzymes, serum proteins, and polypeptide hormones [60]. Optimal dietary Lys supplementation enhances protein deposition and consequently promotes growth performance in ducks. Extensive research indicates that adequate Lys intake increases breast meat yield and body weight gain while reducing feed-to-gain ratio in ducks [7, 32, 61]. As dietary digestible Lys levels increase, nitrogen availability is improved, whereas serum uric acid concentration and nitrogen excretion decrease quadratically, indicating enhanced nitrogen utilization efficiency and reduced environmental nitrogen emissions under optimal Lys supplementation [34]. In breeder ducks, increasing dietary Lys significantly improves reproductive performance and egg quality across multiple parameters, including egg production, egg weight, egg mass, feed conversion ratio, hatchability, hatchling weight, and egg quality parameters in Longyan duck breeders aged 19 to 45 weeks [36]. In addition, dietary Lys markedly affects hepatic triglyceride content and expression of the key lipid metabolism-related genes, including peroxisome proliferator-activated receptor α (PPARα) and carnitine palmitoyltransferase 1 A (CPT1A), which may partly explain the observed reduction in abdominal fat [36]. Furthermore, dietary Lys can enhance the intestinal absorption of minerals such as calcium and iron by forming soluble, low-molecular-weight chelates, thereby improving mineral bioavailability [62].

### Threonine

Thr is generally recognized as the third-limiting amino acid in duck diets, following Met and Lys [63]. However, dietary Thr requirement for Pekin ducks has not been specified by the NRC (1994) [4]. Consequently, a series of experiments have been conducted to evaluate the dietary Thr requirements for Pekin ducks (Table 2). It has been shown that Thr requirements of ducks are influenced by multiple factors, including dietary CP level, growth phase, statistical model, and response criteria [38, 40, 41]. For Pekin ducks from 1 to 21 days of age, the Thr requirement was determined to be 0.67% for achieving maximum weight gain [39]. Using a quadratic broken-line model, Jiang et al. [41] reported that Pekin ducks fed diets containing 19% and 22% CP required 0.66% and 0.70% dietary Thr for optimal weight gain, and 0.67% and 0.73% for breast muscle yield, respectively. Further studies estimated dietary Thr requirements to range from 0.81% to 1.00% using quadratic broken-line regression, and 0.90% to 0.98% using quadratic regression for Pekin ducks from hatch to 14 d of age [37], whereas lower values (0.62% to 0.72% by quadratic broken-line regression, and 0.70% to 0.80% by quadratic regression) were reported for Pekin ducks from 15 to 35 d of age [42]. In Longyan laying ducks (17 to 45 weeks of age), optimal dietary Thr requirements were estimated to be 0.57% for egg production, egg mass, and feed conversion ratio, and 0.58% for egg weight, based on a quadratic broken-line regression model [43]. Clearly, these findings demonstrate that dietary Thr requirements increase with elevating CP levels in the diet [40, 41], are higher for optimizing breast muscle yield than for body weight gain [41], and are generally greater when estimated using quadratic polynomial regression than when derived from linear or quadratic broken-line models [37, 42].

Thr plays a critical role in regulating growth and development in poultry. Numerous studies have demonstrated that dietary Thr supplementation significantly improves growth performance, including improved feed efficiency and increased body weight gain, in starter and growing Pekin ducks [37, 39, 42]. Notably, the growth-promoting effects of Thr are strongly influenced by dietary CP levels and the balance among amino acids [38, 40, 41]. Additionally, adequate Thr supply is essential for maintaining intestinal integrity and promoting intestinal mucin secretion in birds [64, 65]. Xie et al. [39] showed that ducks whose diets were supplemented with 0.74% Thr had greater intestinal villus height than those fed a 0.5% Thr diet. This effect, however, was not observed by Zhang et al. [42], who found that dietary Thr supplementation had no significant impact on villus height or crypt depth in the duodenum and ileum of ducks from 15 to 35 day of age. Mucin 2 (MUC2), produced by goblet cells, constitutes a major component of the intestinal mucus layer and serves as a critical barrier against physical and bacterial damage [66]. Increasing dietary Thr levels from 0.33% to 0.82% significantly upregulated intestinal *MUC2* gene expression by 37% in Pekin ducks [64]. Beyond intestinal function, Thr is also involved in hepatic lipid metabolism. Dietary Thr and CP levels interactively regulate lipid metabolism in the liver of Pekin ducks [38, 41], and low-protein diets have been consistently associated with increased abdominal fat deposition [11, 14, 41]. Appropriate Thr supplementation in low-protein diets reduces hepatic lipid accumulation by decreasing amino acid catabolism and limiting amino acid-to-lipid conversion through improved amino acid balance [41]. Similarly, optimal dietary Thr supplementation has been shown to reduce subcutaneous fat and abdominal fat yield, while increasing breast meat yield [67]. Transcriptome analysis showed dietary Thr deficiency disrupted the proliferation and differentiation of satellite cells in breast muscle [68], and alters the expression of genes involved in lipid metabolism in adipose tissues of Pekin ducks [69]. More recent evidence suggests that Thr deficiency increases triglyceride deposition by reducing signal transducer and activator of transcription 3 (STAT3) phosphorylation [70, 71] and regulating STAT3-stearoyl-CoA desaturase 1 (SCD1) pathway [72] in the liver of Pekin duck or primary duck hepatocyte.

### Tryptophan

Tryptophan (Trp) is an indispensable amino acid that play a crucial role in the growth and development of poultry. According to the NRC (1994) [4], the recommended dietary Trp requirements for Pekin ducks are 0.23% from 0 to 2 weeks of age, 0.17% from 2 to 7 weeks of age, and 0.14% for laying breeders (Table 3). Given the substantial genetic progress and continued performance enhancements in Pekin ducks over recent decades, these recommendations warrant reevaluation (Table 2). Trp exhibits niacin-sparing activity, as it serves as a metabolic precursor for niacin synthesis. A study was therefore conducted to determine the Trp requirement of Pekin ducks from 1 to 21 days of age, demonstrating that ducks fed diets without nicotinamide supplementation required a higher Trp level (0.24%) than those receiving nicotinamide-supplemented diets (0.17%) [44]. In laying ducks, dietary Trp levels critically affected productive performance, egg quality, reproductive organ and ovarian follicle development, and serum biochemical indices, with an optimal dietary Trp range of 0.29% to 0.31% identified for layer breeders during the laying period [45].

The functional roles of dietary Trp in poultry have been extensively reviewed and encompass key physiological processes, including hormone secretion, immune organ development, meat and egg production, and product quality [73]. In Pekin ducks reared under high-density conditions, optimal Trp supplementation (0.48% to 0.78%) enhances growth performance by improving weight gain and feed efficiency [74]. A study by Liu et al. [75] provided mechanistic insight, showing that 5-hydroxytryptophan is localized within the cerebellar Purkinje cell layer of ducks, and that Trp supplementation at 100 to 200 mg/kg significantly increases serotonin (5-HT) levels, a key neuromodulator in regulating feed intake and behavior. However, excessive dietary Trp may competitively inhibit the transport of other neutral amino acids, impairing their absorption and ultimately reducing production performance [73]. Besides, studies have revealed a close association between feather pecking and reduced levels of 5-hydroxytryptophan (a serotonin precursor) in ducks, with lower concentrations consistently observed in individuals exhibiting this behavior [76]. Consistently, Trp depletion models have been shown to promote increased feather pecking. In summary, Trp and its metabolites are essential for improving duck growth, production performance, immune function, and stress resistance. Beyond these established benefits, further studies are needed to define optimal Trp levels in low-protein diets and to elucidate its regulatory mechanisms in duck metabolism.

### Arginine

Arginine (Arg) is an essential amino acid with vital physiological functions in poultry growth and metabolism. However, information regarding Arg requirements in Pekin ducks remains limited. The NRC (1994) [4] recommends levels of 1.1% for ducks from 0 to 2 weeks of age and 1.0% from 2 to 7 weeks of age, but these values were extrapolated from studies conducted in Mule ducks (Table 3). Consequently, Arg requirements for modern Pekin ducks during 1 to 21 days of age have been reevaluated. Dietary Arg supplementation significantly enhanced weight gain, feed intake, and breast meat yield, while reducing the feed-to-gain ratio [46]. Based on quadratic broken-line regression analysis, the Arg requirements for male Pekin ducks during this period were estimated to be 0.95% for maximizing weight gain, 1.16% for optimizing feed-to-gain ratio, and 0.99% for maximizing breast meat yield [46]. In laying ducks, dietary Arg supplementation increased egg weight by enhancing shell weight and yolk deposition, and improved the development of secondary yolk follicles (SYFs) [47]. Regression analyses further indicated that the dietary Arg requirements for Longyan laying ducks aged 17 to 31 weeks were 1.06% for maximizing yolk percentage, 1.13% for maximizing SYFs number, 1.22% for SYFs weight, and 1.11% for the SYFs-to-ovarian weight ratio [47]. The interaction between dietary Arg and Lys has been systematically reviewed in modern poultry nutrition [77]. An inappropriate Arg/Lys ratio can negatively affect growth performance, feed utilization, and plasma and muscle amino acid profiles, with adverse effects being more pronounced under conditions of excessive Lys intake (i.e., a low Arg/Lys ratio). In contrast, excessive Arg (a high Arg/Lys ratio) exerts comparatively weaker negative effects. Our previous analysis showed that an Arg/Lys ratio ranging from 0.86 to 1.55 did not significantly influence the growth performance or muscle yield in Pekin ducks from 1 to 21 days of age [46].

Previous studies have reported that Arg enhances breast muscle yield in broilers [78] and growing ducks [79], and stimulates protein synthesis in the longissimus muscle via the mTOR pathway in neonatal pigs [80]. Consistent with these findings, our study in Pekin ducks from 15 to 35 days of age demonstrated that increasing dietary Arg levels (0.60%, 0.85%, and 1.70%) progressively improved breast muscle yield, feed intake, and carcass yield [81]. Additionally, Arg supplementation has been shown to reduce fat deposition and abdominal adipocyte size in ducks [79]. Under daily cyclic high-temperature conditions, dietary Arg further improved the feed conversion ratio, increased liver weight relative to body weight, and decreased hepatic water content [82]. Dietary Arg deficiency has been reported to impair appetite and growth in broilers [78, 83], a phenomenon that we also observed in Pekin ducks, in which insufficient Arg intake reduced feed intake by up to 39% during the 1 to 21-day period [46, 81]. To elucidate the mechanism underlying Arg-induced regulation of feed intake, attention has focused on its role as a precursor for nitric oxide (NO) synthesis. Rodent studies have demonstrated that inhibition of NO production using L-NAME attenuates the orexigenic effects of ghrelin, orexin A, and morphine [84, 85]. We therefore investigated the relationship between dietary Arg, plasma NO concentration, and feed intake in ducks [86]. Ducks fed a low-Arg diet (0.65%) exhibited reduced feed intake and plasma NO levels compared with those fed diets containing 0.95% or 1.45% Arg, supporting the conclusion that Arg modulates feeding behavior in Pekin ducks, likely through regulation of endogenous NO synthesis.

### Other amino acids

The shift toward low-protein and low-soybean-meal diets in duck production requires increased consideration of other amino acids, such as branched-chain amino acids (leucine (Leu), isoleucine (Ile), valine (Val)), serine (Ser), and glycine (Gly). As reviewed by Kim et al. [87], branched-chain amino acids play critical functional roles in various poultry species, including broilers, laying hens, turkeys, and quails, but their requirement and optimal ratios in ducks are poorly defined. One of the few studies addressing this issue, conducted by Timmler and Rodehutscord [88], identified a dietary Val level of 0.70% as optimal for protein accretion in Pekin ducks, although no significant effects on growth performance were observed. Nevertheless, this value was lower than the NRC (1994) [4] recommendation (Table 3). Recently, Huang et al. [89] reported that a total dietary branched-chain amino acid level of 3.76%, with a Leu:Val:Ile ratio ranging from 1:0.6:0.5 to 1:0.7:0.6, optimized growth performance and serum biochemical indices in Youxian partridge laying ducks from 29 to 63 days of age. Although serine and glycine are traditionally classified as non-essential amino acids, they perform indispensable physiological functions in poultry by supporting protein synthesis and serving as key precursors for metabolites involved in multiple biological pathways [90]. Despite the lack of glycine dose–response studies in poultry other than broilers, work in White Pekin ducks demonstrates that free glycine supplementation allows for dietary CP reduction in progressively diluted diets without impairing growth performance, when essential amino acid levels are adequate [14].

### The variability of estimated amino acid requirements

Although numerous studies have contributed valuable data on amino acid requirements in ducks, considerable variability exists among reported estimates, which is partially attributed to methodological differences. One major source of inconsistency arises from the statistical models used to estimate requirements. Quadratic regression models typically yield higher requirement values by optimizing biological responses [24, 25, 34], whereas linear or quadratic broken-line models tend to provide more conservative estimates by identifying minimal levels required to achieve a response plateau [6, 52, 81]. The actual nutritional requirement is generally considered to fall between these two estimates. The intersection point of the broken-line and quadratic regression model has been proposed as a more biologically meaningful and practically applicable indicator of amino acid requirements [91, 92]. Besides, variations in experimental design further contribute to discrepancies among studies. Differences in basal diet composition, particularly crude protein level and amino acid balance, can influence the estimated requirement of a target amino acid through nutrient interactions [6, 38]. Moreover, genetic strain, age, sex, and housing system (e.g., floor vs. cage rearing) affect growth potential, feed intake patterns, and nutrient utilization efficiency, altering their amino acid requirements. The choice of response criteria also plays a critical role; requirements based on average daily gain or feed efficiency may differ from those derived from carcass traits such as breast muscle yield or reproductive performance [32, 81].

## The current amino acid requirement guidelines for ducks

For decades, the NRC (1994) [4] has provided reference values for amino acid requirements in ducks (Table 3) and continues to serve as a guiding standard for diet formulation and a fundamental basis for assessing dietary nutrient adequacy. However, the duck industry has undergone substantial genetic, nutritional, and managerial advancements over recent decades, accompanied by pronounced shifts in rearing systems and feeding strategies, including the widespread adoption of cage housing and low-protein, low-soybean-meal diversified diets. These developments have prompted extensive research aimed at redefining the amino acid requirements of modern meat and laying ducks to better align nutritional supply with contemporary production systems. Based on these scientific advances, China has officially issued two national feeding standards for duck nutrition, namely *Nutrient Requirements of Meat-type Ducks* (GB/T 45103–2024) [93] and *Nutrient Requirements of Egg Ducks* (GB/T 41189–2021) [94]. The standard for meat-type ducks systematically specifies nutrient specifications for Pekin ducks, Muscovy ducks, and meat-egg dual-purpose ducks across starter, grower, finisher, and breeder phases, whereas the standard for egg-laying ducks defines the specific nutrient requirements of small-, and medium-sized laying duck strains. These standards provide comprehensive nutritional specifications for meat ducks (Tables 4 and 5) and egg-laying ducks (Table 6), including dietary energy, CP, major amino acids expressed as both total and standardized digestible values, as well as key nutritional ratios such as CP/ME and Lys/ME. Viewed in relation to the long-standing NRC (1994) [4] framework, these updated standards offer complementary, evidence-based refinements that reflect the nutritional demands of modern duck genotypes and may inform future updates of global duck nutrient requirement models.

**Table 4:** Dietary energy, crude protein and amino acid requirements of commercial meat-type ducks^a^^,b^

Nutrients^c^	Pekin duck				Muscovy duck			Meat-egg dual-purpose duck		
	Starter (0–2 weeks)	Grower (3–5 weeks)	Finisher (6 weeks ~)		Starter (0–3 weeks)	Grower (4–7 weeks)	Finisher (8 weeks to slaughter)	Starter (0–3 weeks)	Grower (4–7 weeks)	Finisher (8 weeks to slaughter)
			Ad libitum	Over-feeding						
AME, MJ/kg (kcal/kg)	11.93 (2,850)	12.14 (2,900)	12.35 (2,950)	12.56 (3,000)	11.93 (2,850)	11.72 (2,800)	11.72 (2,800)	11.72 (2,800)	11.30 (2,700)	11.30 (2,700)
EHGE, MJ/kg (kcal/kg)	12.35 (2,950)	12.56 (3,000)	12.77 (3,050)	12.98 (3,100)	12.35 (2,950)	12.14 (2,900)	12.14 (2,900)	12.14 (2,900)	11.72 (2,800)	11.72 (2,800)
CP, %	19.5	17.5	16.0	13.0	19.0	16.5	14.5	19.0	17.0	14.0
CP/AME, g/MJ (g/Mcal)	16.3 (68.4)	14.4 (60.3)	13.0 (54.2)	10.4 (43.3)	15.9 (66.7)	14.1 (58.9)	12.4 (51.8)	16.2 (67.9)	15.0 (63.0)	12.4 (51.9)
Lys/AME, g/MJ (g/Mcal)	0.92 (3.86)	0.70 (2.93)	0.57 (2.37)	0.48 (2.00)	0.88 (3.68)	0.68 (2.86)	0.55 (2.32)	0.90 (3.75)	0.75 (3.15)	0.58 (2.41)
Total amino acids, %										
Lys	1.10	0.85	0.70	0.60	1.05	0.80	0.65	1.05	0.85	0.65
Met	0.45	0.40	0.35	0.30	0.45	0.40	0.35	0.40	0.38	0.35
Met + Cys	0.82	0.72	0.65	0.55	0.80	0.75	0.60	0.78	0.70	0.60
Thr	0.72	0.60	0.55	0.47	0.75	0.60	0.45	0.75	0.60	0.50
Trp	0.20	0.18	0.16	0.14	0.20	0.18	0.16	0.18	0.16	0.14
Ile	0.72	0.57	0.45	0.40	0.70	0.55	0.50	0.70	0.55	0.50
Arg	1.00	0.85	0.70	0.60	0.90	0.80	0.65	0.90	0.80	0.70
True available amino acids, %										
TA-Lys	0.98	0.76	0.60	0.53	0.94	0.71	0.57	0.93	0.75	0.57
TA-Met	0.42	0.37	0.32	0.28	0.42	0.37	0.32	0.37	0.35	0.32
TA-Met + Cys	0.72	0.64	0.58	0.49	0.71	0.67	0.55	0.69	0.62	0.55
TA-Thr	0.62	0.52	0.48	0.40	0.65	0.52	0.39	0.65	0.52	0.43
TA-Trp	0.19	0.17	0.15	0.13	0.19	0.17	0.15	0.17	0.15	0.13
TA-Ile	0.65	0.50	0.39	0.34	0.61	0.48	0.43	0.61	0.47	0.43
TA-Arg	0.95	0.80	0.66	0.57	0.85	0.75	0.61	0.85	0.76	0.66

^a^The data were obtained in accordance with the Chinese National Standard “*Nutrient Requirements of Meat-type Ducks*” (GB/T 45103–2024) [93]

^b^The ducks were fed ad libitum, with all data calculated on the basis of 88% dietary dry matter

^c^*AME* Apparent metabolizable energy, *EHGE* Enzymatic hydrolyzate gross energy, *CP* Crude protein, *Met* Methionine, *Cys* Cysteine, *Lys* Lysine, *Thr* Threonine, *Trp* Tryptophan, *Arg* Arginine, *Ile* Isoleucine, *TA* True available

**Table 5:** Dietary energy, crude protein and amino acid requirements of meat-type duck breeders^a^^,b^

Nutrients^c^	Pekin duck						Muscovy duck				Meat-egg dual-purpose duck					
	Starter (0–3 weeks)	Grower (4–8 weeks)	Grower (9–22 weeks)	Layer (23–26 weeks)	Layer (27–45 weeks)	Layer (46–75 weeks)	Starter (0–3 weeks)	Grower (4–7 weeks)	Grower (8–26 weeks)	Layer (27–65 weeks)	Starter (0–3 weeks)	Grower (4–7 weeks)	Grower (8–18 weeks)	Layer (19–22 weeks)	Layer (23–45 weeks)	Layer (46–75 weeks)
AME, MJ/kg (kcal/kg)	11.93 (2,850)	11.93 (2,850)	11.30 (2,700)	11.72 (2,800)	11.51 (2,750)	11.30 (2,700)	11.72 (2,800)	11.72 (2,800)	11.30 (2,700)	11.30 (2,700)	11.72 (2,800)	11.30 (2,700)	11.30 (2,700)	11.09 (2,650)	11.09 (2,650)	11.09 (2,650)
EHGE, MJ/kg (kcal/kg)	11.93 (2,850)	11.93 (2,850)	11.30 (2,700)	12.14 (2,900)	11.93 (2,850)	11.72 (2,800)	12.14 (2,900)	12.14 (2,900)	11.72 (2,800)	11.72 (2,800)	12.14 (2,900)	11.72 (2,800)	11.72 (2,800)	11.51 (2,750)	11.51 (2,750)	11.51 (2,750)
CP, %	19.5	17.5	15.0	17.0	18.0	18.5	19.0	17.0	14.0	17.0	19.0	17.0	14.0	16.0	17.0	17.0
CP/AME, g/MJ (g/Mcal)	16.3 (68.4)	14.7 (61.4)	13.3 (55.6)	14.5 (60.7)	15.6 (65.5)	16.4 (68.5)	16.2 (67.9)	14.5 (60.7)	12.4 (51.9)	15.0 (63.0)	16.2 (67.9)	15.0 (63.0)	12.4 (51.9)	14.4 (60.4)	15.3 (64.2)	15.3 (64.2)
Lys/AME, g/MJ (g/Mcal)	0.88 (3.68)	0.71 (2.98)	0.58 (2.41)	0.68 (2.86)	0.83 (3.45)	0.88 (3.70)	0.90 (3.75)	0.68 (2.86)	0.53 (2.22)	0.75 (3.15)	0.85 (3.57)	0.71 (2.96)	0.58 (2.41)	0.72 (3.02)	0.77 (3.21)	0.77 (3.21)
Total amino acids, %																
Lys	1.05	0.85	0.65	0.80	0.95	1.00	1.05	0.80	0.60	0.85	1.00	0.80	0.65	0.80	0.85	0.85
Met	0.45	0.40	0.35	0.40	0.45	0.45	0.45	0.40	0.30	0.40	0.40	0.35	0.30	0.38	0.38	0.40
Met + Cys	0.80	0.70	0.60	0.70	0.75	0.75	0.80	0.75	0.55	0.72	0.78	0.70	0.60	0.65	0.70	0.72
Thr	0.75	0.60	0.50	0.60	0.65	0.70	0.75	0.60	0.45	0.60	0.70	0.60	0.50	0.60	0.60	0.65
Trp	0.20	0.18	0.16	0.20	0.20	0.22	0.20	0.18	0.16	0.18	0.20	0.18	0.16	0.20	0.18	0.20
Ile	0.72	0.55	0.45	0.57	0.68	0.72	0.70	0.55	0.42	0.68	0.68	0.55	0.40	0.55	0.65	0.65
Arg	0.95	0.80	0.70	0.90	0.90	0.95	0.90	0.80	0.65	0.80	0.95	0.80	0.65	0.80	0.85	0.85
True available amino acids, %																
TA-Lys	0.94	0.76	0.57	0.71	0.84	0.89	0.94	0.71	0.53	0.76	0.89	0.71	0.57	0.71	0.75	0.75
TA-Met	0.42	0.37	0.32	0.37	0.42	0.42	0.42	0.37	0.27	0.37	0.37	0.32	0.27	0.35	0.35	0.37
TA-Met + Cys	0.73	0.62	0.55	0.63	0.67	0.67	0.71	0.67	0.49	0.64	0.70	0.63	0.54	0.58	0.63	0.64
TA-Thr	0.66	0.52	0.43	0.52	0.57	0.61	0.65	0.52	0.39	0.52	0.61	0.52	0.43	0.52	0.52	0.56
TA-Trp	0.19	0.17	0.15	0.19	0.19	0.21	0.19	0.17	0.15	0.17	0.19	0.17	0.15	0.19	0.17	0.19
TA-Ile	0.65	0.48	0.38	0.49	0.59	0.62	0.61	0.48	0.36	0.59	0.59	0.47	0.34	0.47	0.56	0.56
TA-Arg	0.90	0.75	0.66	0.85	0.85	0.90	0.85	0.75	0.61	0.76	0.90	0.76	0.61	0.76	0.80	0.80

^a^The data were obtained in accordance with the Chinese National Standard “*Nutrient Requirements of Meat-type Ducks*” (GB/T 45103–2024) [93]

^b^The ducks were fed ad libitum, with all data calculated on the basis of 88% dietary dry matter

^c^*AME* Apparent metabolizable energy, *EHGE* Enzymatic hydrolyzate gross energy, *CP* Crude protein, *Met* Methionine, *Cys* Cysteine, *Lys* Lysine, *Thr* threonine, *Trp* Tryptophan, *Arg* Arginine, *Ile* Isoleucine, *TA* True available

**Table 6:** Dietary energy, crude protein and amino acid requirements for egg-laying ducks^a^^,b^

Nutrients^c^	Small-sized egg-laying duck						Medium-sized egg-laying duck					
	0–4 weeks	5–12 weeks	13 weeks – 5% LR	Layer (5% < LR < 80%)	Layer (LR ≥ 80%)	Layer (LR < 80%)	0–4 weeks	5–12 weeks	13 weeks – 5% LR	Layer (5% < LR < 80%)	Layer (LR ≥ 80%)	Layer (LR < 80%)
AME, MJ/kg (kcal/kg)	11.72 (2,800)	10.88 (2,600)	10.88 (2,600)	10.46 (2,500)	10.46 (2,500)	10.46 (2,500)	11.9 (2,850)	11.3 (2,700)	11.3 (2,700)	10.67 (2,550)	10.67 (2,550)	10.67 (2,550)
CP, %	18.5	16.0	14.0	16.0	16.5	17.0	19.0	16.0	14.0	16.5	17.0	17.5
CP/AME, g/MJ (g/Mcal)	15.8 (66.1)	14.7 (61.5)	12.9 (53.8)	15.3 (64.0)	15.8 (66.0)	16.3 (68.0)	16.0 (66.7)	14.2 (59.3)	12.4 (51.9)	15.5 (64.7)	15.9(66.7)	16.4 (68.6)
Lys/AME, g/MJ (g/Mcal)	0.85 (3.57)	0.78 (3.27)	0.64 (2.69)	0.76 (3.20)	0.81 (3.40)	0.84 (3.52)	0.85 (3.54)	0.75 (3.15)	0.66 (2.78)	0.82 (3.41)	0.84 (3.53)	0.87 (3.65)
Total amino acids, %												
Lys	1.00	0.85	0.70	0.80	0.85	0.88	1.01	0.85	0.75	0.87	0.90	0.93
Met	0.42	0.40	0.30	0.40	0.40	0.41	0.45	0.40	0.30	0.40	0.40	0.41
Met + Cys	0.76	0.68	0.56	0.68	0.68	0.70	0.78	0.68	0.56	0.68	0.69	0.70
Thr	0.70	0.60	0.53	0.60	0.55	0.57	0.71	0.60	0.53	0.63	0.65	0.67
Trp	0.20	0.18	0.16	0.19	0.20	0.21	0.21	0.18	0.16	0.19	0.20	0.21
Ile	0.64	0.55	0.48	0.63	0.65	0.67	0.65	0.55	0.48	0.63	0.65	0.67
Arg	0.90	0.80	0.70	0.87	0.90	0.93	0.92	0.80	0.70	0.87	0.90	0.93
True available amino acids, %												
TA-Lys	0.92	0.76	0.62	0.70	0.76	0.80	0.90	0.76	0.66	0.77	0.80	0.83
TA-Met	0.39	0.39	0.27	0.34	0.37	0.39	0.40	0.37	0.27	0.37	0.37	0.38
TA-Met + Cys	0.67	0.60	0.49	0.61	0.61	0.63	0.68	0.59	0.48	0.61	0.62	0.63
TA-Thr	0.62	0.51	0.47	0.53	0.49	0.50	0.63	0.53	0.48	0.54	0.56	0.59
TA-Trp	0.19	0.17	0.15	0.18	0.19	0.20	0.20	0.17	0.15	0.18	0.19	0.20
TA-Ile	0.54	0.47	0.41	0.54	0.56	0.57	0.55	0.47	0.41	0.53	0.55	0.57
TA-Arg	0.82	0.73	0.64	0.80	0.89	0.85	0.84	0.73	0.65	0.80	0.81	0.85

^a^The data were obtained in accordance with the Chinese National Standard “*Nutrient Requirements of Egg Ducks*” (GB/T 41189–2021) [94]

^b^The ducks were fed ad libitum, with all data calculated on the basis of 88% dietary dry matter

^c^*AME* Apparent metabolizable energy, *CP* Crude protein, *Met* Methionine, *Cys* Cysteine, *Lys* Lysine, *Thr* Threonine, *Trp* Tryptophan, *Arg* Arginine, *Ile* Isoleucine, *TA* True available, *LR* Laying rate

## Summary

Clearly, the NRC (1994) recommendations are insufficient for contemporary, high-performing duck strains. This review summarizes updated estimates for the requirements of CP and key amino acids, such as Met, Lys, Thr, Trp, and Arg, which are essential for formulating precision diets to maximize growth performance, carcass yield, and egg production. Beyond their conventional role as protein constituents, these amino acids serve critical regulatory functions in multiple physiological pathways, including lipid metabolism (Met, Thr), intestinal barrier integrity (Thr), feed intake regulation via nitric oxide (Arg), and modulation of behavior and stress responses (Trp). The adoption of low-protein diets (15% CP for growing meat ducks, 14.5% CP for laying ducks) improves nitrogen use efficiency, reduces nitrogen emissions, and decreases reliance on soybean meal in duck production. The recently published Chinese national feeding standards for meat-type and laying ducks incorporate these research advances, providing a modernized framework to guide the global duck industry. Future research should define precise requirements for branched-chain amino acids (Val, Ile, Leu) and certain non-essential amino acids (Gly, Ser) in ducks. Understanding how amino acids regulate gene expression, signaling pathways, and gut microbiota will enable more precise nutritional management. Combining low-protein diet formulation with the use of unconventional feed resources can promote soybean meal reduction and substitution. Together, these strategies are essential for improving growth performance, nutrient efficiency, and sustainability in duck production.

## Data Availability

Not applicable.

## Abbreviations

ADG: Average daily gain
Arg: Arginine
BHMT: Betaine-homocysteine methyltransferase
Cys: Cysteine
CP: Crude protein
CPT1A: Carnitine palmitoyltransferase 1A
F/G: Feed-to-gain ratio
FCR: Feed conversion ratio
Gly: Glycine
GPX: Glutathione peroxidase
GSH: Glutathione
HMTBA: 2-Hydroxy-4-methylthiobutanoic acid
Ile: Isoleucine
Leu: Leucine
Lys: Lysine
MDA: Malondialdehyde
ME: Metabolizable energy
Met: Methionine
MS: Methionine synthase
mTOR: Mammalian target of rapamycin
MUC2: Mucin 2
NO: Nitric oxide
NRC: National Research Council
Nrf2: Nuclear factor erythroid 2-related factor 2
PPARα: Peroxisome proliferators-activated receptor alpha
SCD1: Stearoyl-CoA desaturase 1
Ser: Serine
STAT3: Signal transducer and activator of transcription 3
SYF: Secondary yolk follicle
TG: Triglyceride
Thr: Threonine
Trp: Tryptophan
Val: Valine
